# Post-marketing safety of lorlatinib: a real-world study based on the FDA adverse event reporting system

**DOI:** 10.3389/fphar.2024.1385036

**Published:** 2024-06-05

**Authors:** Huqun Li, Chongshu Wang, Cuilian Guo

**Affiliations:** ^1^ Department of Pharmacy, The Central Hospital of Wuhan, Tongji Medical College, Huazhong University of Science and Technology, Wuhan, China; ^2^ Department of Otolaryngology-Head and Neck Surgery, Tongji Hospital, Tongji Medical College, Huazhong University of Science and Technology, Wuhan, China

**Keywords:** lorlatinib, pharmacovigilance, disproportionality, adverse event, FAERS

## Abstract

**Background:**

Lorlatinib displays marked systemic and intracranial efficacy against anaplastic lymphoma kinase (ALK) positive non-small cell lung cancer (NSCLC). We aimed to establish the safety profile of lorlatinib based on the Food and Drug Administration Adverse Event Reporting System (FAERS).

**Methods:**

Reports from the FAERS between 2019 and 2023 were collected to conduct the disproportionality analysis. Reporting odds ratio (ROR) was employed to detect the potential adverse events (AEs) related to lorlatinib. The clinical characteristics, age and gender differences, time to onset of AEs were also investigated.

**Results:**

A total of 2,941 AE reports were found to be associated with lorlatinib among the 8,818,870 AE reports obtained from the FAERS database. 167 lorlatinib-related AE signals were identified. The frequently reported AEs including hypercholesterolemia, oedema, and cognitive disorder were in line with those observed in clinical trials and drug instruction. However, AEs such as interstitial lung disease and AV block indicated in the drug label require further evaluation. More attention should be paid to the new potential unexpected AEs including pulmonary arterial hypertension and radiation necrosis. Furthermore, we examined the specific high-risk AEs of different ages and genders. In addition, majority of AEs occurred within the first 2 months after lorlatinib initiation with a median onset time of 51 days.

**Conclusion:**

Our study provides valuable insight into the post-marketing safety profile of lorlatinib, which can potentially benefit the rational and safe administration of lorlatinib in the clinic. Further prospective studies are needed to validate the associations between lorlatinib and the identified AEs.

## Introduction

Lung cancer accounts for 13% of total cancer-related deaths globally and has become the leading cause of cancer-related mortality (Ando et al., 2021). Non-small cell lung cancer (NSCLC) that was often complicated with brain metastases accounted for 85%–90% of all lung cancers (Yang and Gong, 2019). Approximately 3%–5% of patients with NSCLC belonged to anaplastic lymphoma kinase (ALK)-positive NSCLC which provided opportunities for targeted therapy (Chen et al., 2021). The first- and second-generation ALK-tyrosine kinase inhibitors (TKIs) such as crizotinib and alectinib have shown promising efficacy against ALK-positive NSCLC. However, these therapies have been frequently hampered by the acquired drug resistance and progression of central nervous system (CNS) metastases (Reed et al., 2020).

As a third-generation ALK-TKI, lorlatinib was designed and developed with good penetration of the blood-brain barrier and broad-spectrum potency against most known resistance mutations during treatment with first- and second-generation ALK-TKIs (Reed et al., 2020). Lorlatinib has shown marked systemic and intracranial activity in patients with ALK-positive advanced NSCLC who were treatment-naive or who had progressed on a range of prior ALK-TKIs (Shaw et al., 2017; Shaw et al., 2020). Lorlatinib has been approved by Food and Drug Administration (FDA) in November 2018 and later by European Medicines Agency (EMA) in March 2019. Despite being generally well tolerated, lorlatinib has a unique and challenging safety profile compared with other ALK-TKIs that is characterized by hyperlipidemia, cognitive effects, mood effects, oedema, and peripheral neuropathy which are documented by both FDA and EMA. Uncommon yet serious adverse events (AEs) including pneumonitis have been reported as well (Harrison et al., 2022). Lorlatinib-related AEs are predominantly mild to moderate in severity and could be effectively managed with dose modification and standard supportive therapy. However, permanent discontinuation due to AEs including cognitive disorder and mood disorder occurred in 2%–7% of the patients in previous studies (Bauer et al., 2019; Shaw et al., 2020). Moreover, most evidence of lorlatinib-related AEs has been generally from clinical studies with strict inclusion criteria and limited sample sizes and case reports. With the increasing use of lorlatinib in clinical practice, a systemic and comprehensive evaluation of the safety profile of lorlatinib in real-world settings is urgently required.

The FDA Adverse Event Reporting System (FAERS) is a publicly accessible database that collects AE reports worldwide to support the post-marketing safety monitoring of drugs and therapeutic biologic products. In a recent post-marketing safety study of ALK inhibitors, metabolism disorders and neoplasms on the system organ class level and hypercholesterolemia and pulmonary arterial hypertension on the preferred term level were identified for lorlatinib (Omar et al., 2021). However, a detailed safety profile and early detection of new potential AEs for lorlatinib are still lacking. In the present study, we performed a pharmacovigilance analysis of lorlatinib based on the FAERS database and aimed to provide a thorough evaluation of lorlatinib-related AEs in real-world settings and promote the rational and safe use of lorlatinib in clinic.

## Materials and methods

### Data sources

The data of this pharmacovigilance study were collected from the FAERS from 2019 to 2023. Based on the FAERS, seven data files were provided: DEMO (demographic and administrative information), DRUG (information for drug administration), REAC (adverse event coded by preferred terms), OUTC (outcomes of patients), THER (therapy start dates and end dates for reported drugs), INDI (indications for drugs), and PRSR (report sources). These files were linked through the PRIMARYID field in each file. As the reports in FAERS were submitted spontaneously by healthcare professionals (physicians, pharmacists) and non-healthcare professionals (lawyers, consumers), there inevitably were duplicate reports. Following the FDA’s suggestions, we chose the latest FDA_DT when the PRIMARYIDs were the same, and chose the higher PRIMARYID when the FDA_DT and the CASEID were the same, to conduct de-duplication before data analysis. Both generic name (lorlatinib) and brand name (lorbrena) were used to obtain a comprehensive accounting of lorlatinib-associated reports. Only reports in which lorlatinib was considered the primary suspect (PS) drug with the ROLE_COD being “PS” were collected to improve accuracy. AEs in the FAERS database are coded by the preferred term (PT) and subsequently classified to the corresponding system organ classes (SOCs) based on the standardized Medical Dictionary for Regulatory Activities (MedDRA) terminology (version 26.1). Available information on age, sex, indication, reporter country, and patient outcomes was documented to characterize lorlatinib-related AEs in our analysis. Subgroup analysis was performed to investigate the potential difference in safety profiles of lorlatinib in subgroups based on age [aged younger than 18 (pediatric) and aged 18 and older (adult)] and gender (male and female). The onset time was calculated from the onset date of AE (EVENT_DT field in the DEMO file) minus the start date of lorlatinib treatment (START_DT field in the THER file). Reports with input errors (inaccurate entries, missing data, and EVENT_DT earlier than START_DT) were excluded.

### Statistical analysis

In the current pharmacovigilance study, disproportionality analysis was applied to detect potential safety signals for lorlatinib. Reporting odds ratio (ROR) was calculated by standard formulas (Table 1) to investigate the associations between lorlatinib and AEs. Safety signals were considered significant when the lower border of 95% confidence interval exceeded 1.0 with at least 3 AE reports. *p*-value <0.05 was considered statistically significant. Data mining and statistical analysis were conducted by MYSQL 8.0 and Navicat Premium 15, SPSS 29.0, and Microsoft Excel 2019.

**TABLE 1:** Calculation of ROR and 95% CI.

Drug category	Event of interest	All other events
Lorlatinib	a	b
All other drugs	c	d

ROR, ad/bc; 95% CI, e^ln(ROR)±1.96(1/a+1/b+1/c+1/d)^0.5^; ROR, reporting odds ratio; CI, confidence interval.

## Results

### Descriptive analysis

In the present study, 2941 lorlatinib-related reports were obtained from 8,181,870 AE reports collected from the FAERS database during the study period. The clinical characteristics of these reports were illustrated in Table 2. Females and males accounted for 45.70% and 37.47%, respectively. Lorlatinib-related AEs were more likely to occur in patients aged 18 years and older (65.62%). America submitted the most reports (39.65%), followed by Japan (13.60%), India (12.55%), France (4.32%), and Canada (3.91%). The most reported indication was lung neoplasm malignant (13.74%), followed by non-small cell lung cancer (13.33%), lung adenocarcinoma (3.06%), non-small cell lung cancer metastatic (2.86%), and lung cancer metastatic (2.52%), which was in line with the drug instructions. However, off-label uses of lorlatinib were also commonly reported such as neuroblastoma (1.60%) and adenocarcinoma (0.42%). Regarding the severe outcomes of the identified AEs, other medical events was most frequently reported (35.02%), followed by death (25.98%) and hospitalization (16.12%). AE reports gradually increased from 453 (15.40%) reports in 2019 to 911 (30.98%) reports in 2023.

**TABLE 2:** Clinical characteristics of lorlatinib-related adverse event reports based on the FAERS database (2019–2023).

Characteristics	Case Number, n	Proportion, %
**Number of events**	2941	
**Sex**		
Female	1344	45.70
Male	1102	37.47
Unknown	495	16.83
**Age (years)**		
<18	130	4.42
18≤ and <60	902	30.67
≥60	1028	34.95
Unknown	881	29.96
**Indications (top five)**		
Lung neoplasm malignant	404	13.74
Non-small cell lung cancer	392	13.33
Lung adenocarcinoma	90	3.06
Non-small cell lung cancer metastatic	84	2.86
Lung cancer metastatic	74	2.52
**Serious outcome**		
Death (DE)	764	25.98
Life-threatening (LT)	31	1.05
Hospitalization-initial or prolonged (HO)	474	16.12
Disabled (DS)	22	0.75
Other important medical event (OT)	1030	35.02
Required Intervention (RI)	3	0.10
**Reported countries (top five)**		
America (US)	1166	39.65
Japan (JP)	400	13.60
India (IN)	369	12.55
France (FR)	127	4.32
Canada (CA)	115	3.91
**Reporting year**		
2023	911	30.98
2022	585	19.89
2021	531	18.06
2020	461	15.67
2019	453	15.40

### Signal detection

Signal detection results are present in Table 3 (Detail in Supplementary Table S1). A total of 167 lorlatinib-related safety signals across 20 SOCs were identified by disproportionality analysis. In our study, commonly reported AEs including hypercholesterolemia (PT: 10020603), hypertriglyceridemia (PT: 10020869), oedema (PT: 10030095), and cognitive disorder (PT: 10057668) were identified which was consistent with the drug instructions and clinical studies. New and unexpected AEs that were not included in the drug label were also observed, such as thrombophlebitis migrans (PT: 10043581), radiation necrosis (PT: 10067362), and pericardial effusion (PT: 10034474). Of note, although thrombophlebitis migrans (*n* = 6, ROR 64.80) and radiation necrosis (*n* = 8, ROR 78.00) had small numbers of cases, they showed high signal values, which required further study. Additionally, AEs that are indicated in the label including arthralgia, constipation, and back pain did not meet the criteria for positive signals in our analysis.

**TABLE 3:** Signal strength of lorlatinib related AEs at the PTs level in FAERS database (n ≥ 10).

SOC	PTs	Cases (n)	ROR (95% CI)
Cardiac disorders	Cardiac failure	34	2.75 (1.96–3.86)
	Pericardial effusion	33	9.58 (6.80–13.51)
Ear and labyrinth disorders	Deafness	10	2.26 (1.22–4.21)
	Tinnitus	17	2.67 (1.66–4.31)
Endocrine disorders	Hypothyroidism	16	2.77 (1.70–4.53)
Eye disorders	Blindness	12	1.82 (1.03–3.21)
Gastrointestinal disorders	Ascites	12	2.77 (1.57–4.88)
	Dysphagia	28	2.09 (1.44–3.03)
General disorders and administration site conditions	Death	677	6.83 (6.27–7.44)
	General physical health deterioration	30	1.57 (1.09–2.25)
	Generalised oedema	13	8.38 (4.85–14.45)
	oedema	95	13.06 (10.64–16.03)
	oedema peripheral	101	7.41 (6.07–9.03)
	Peripheral swelling	73	2.13 (1.69–2.69)
Hepatobiliary disorders	Hepatic function abnormal	14	2.34 (1.38–3.96)
Infections and infestations	Pneumonia	70	1.28 (1.01–1.63)
	Pneumonia aspiration	10	2.61 (1.40–4.85)
Investigations	Aspartate aminotransferase increased	15	2.09 (1.26–3.48)
	Blood cholesterol increased	127	19.45 (16.27–23.25)
	Blood creatinine decreased	18	1.72 (1.08–2.73)
	Blood glucose increased	35	1.55 (1.11–2.16)
	Blood thyroid stimulating hormone increased	12	9.45 (5.36–16.68)
	Blood triglycerides increased	66	37.92 (29.67–48.48)
	Body mass index increased	10	36.10 (19.32–67.43)
	Electrocardiogram QT prolonged	26	4.49 (3.05–6.61)
	Gamma-glutamyltransferase increased	14	4.74 (2.80–8.02)
	Glycosylated haemoglobin increased	11	2.88 (1.59–5.21)
	Haemoglobin decreased	43	2.61 (1.93–3.53)
	Lipids abnormal	10	88.58 (47.15–166.43)
	Lipids increased	16	57.13 (34.78–93.85)
	Liver function test increased	12	2.52 (1.43–4.44)
	Low density lipoprotein increased	39	39.47 (28.71–54.25)
	Weight increased	109	2.81 (2.32–3.40)
	White blood cell count increased	13	2.33 (1.35–4.03)
Metabolism and nutrition disorders	Diabetes mellitus	22	1.92 (1.26–2.92)
	Dyslipidaemia	29	33.21 (22.99–47.98)
	Fluid retention	24	2.93 (1.96–4.37)
	Hypercholesterolaemia	91	80.67 (65.29–99.67)
	Hyperglycaemia	14	2.78 (1.64–4.70)
	Hyperlipidaemia	66	62.36 (48.73–79.80)
	Hypertriglyceridaemia	54	64.34 (49.01–84.47)
Neoplasms benign, malignant and unspecified (incl cysts and polyps)	Neoplasm progression	494	67.34 (61.08–74.23)
	Second primary malignancy	17	8.52 (5.28–13.73)
Nervous system disorders	Altered state of consciousness	14	4.43 (2.62–7.49)
	Amnesia	27	3.10 (2.12–4.52)
	Aphasia	19	4.27 (2.72–6.70)
	Brain oedema	21	11.96 (7.78–18.39)
	Carpal tunnel syndrome	12	4.66 (2.64–8.22)
	Cerebral haemorrhage	12	2.59 (1.47–4.57)
	Cerebral infarction	19	6.28 (4.00–9.87)
	Cerebrovascular accident	29	1.54 (1.07–2.22)
	Cognitive disorder	89	11.45 (9.26–14.14)
	Depressed level of consciousness	13	2.29 (1.33–3.96)
	Dysarthria	20	4.16 (2.68–6.46)
	Memory impairment	61	2.48 (1.92–3.20)
	Mental impairment	12	3.44 (1.95–6.06)
	Nervous system disorder	41	14.10 (10.35–19.20)
	Neuropathy peripheral	56	3.35 (2.57–4.36)
	Neurotoxicity	19	5.92 (3.77–9.30)
	Speech disorder	44	5.64 (4.18–7.59)
Psychiatric disorders	Affective disorder	12	9.15 (5.19–16.15)
	Aggression	14	2.68 (1.59–4.53)
	Confusional state	55	2.44 (1.87–3.19)
	Delirium	24	4.81 (3.22–7.20)
	Disorientation	12	2.57 (1.46–4.53)
	Hallucination	110	9.34 (7.72–11.30)
	Hallucination, auditory	34	16.26 (11.58–22.82)
	Hallucination, visual	30	9.04 (6.30–12.95)
	Irritability	15	2.32 (1.39–3.85)
	Mental disorder	32	4.50 (3.18–6.38)
	Mood altered	15	4.31 (2.59–7.16)
	Mood swings	11	3.11 (1.72–5.63)
	Psychiatric symptom	12	10.41 (5.90–18.37)
	Psychotic disorder	33	9.58 (6.80–13.51)
Renal and urinary disorders	Urinary incontinence	12	2.77 (1.57–4.89)
Respiratory, thoracic and mediastinal disorders	Dyspnoea	116	1.30 (1.08–1.57)
	Dyspnoea exertional	14	1.78 (1.05–3.01)
	Haemoptysis	15	3.56 (2.14–5.91)
	Pleural effusion	70	8.28 (6.53–10.49)
	Pneumonitis	28	5.83 (4.02–8.46)
	Pulmonary embolism	42	3.86 (2.85–5.24)
	Pulmonary hypertension	11	3.79 (2.10–6.86)
	Pulmonary oedema	16	2.49 (1.52–4.07)
	Respiratory disorder	13	2.67 (1.55–4.60)
	Respiratory failure	20	2.09 (1.35–3.24)
Vascular disorders	Deep vein thrombosis	16	2.51 (1.53–4.10)

AE, adverse event; PT, preferred term; SOC, system organ class; ROR, reporting odds ratio; CI, confidence interval.

### Subgroup analysis

Among the top 15 AEs, dyspnoea, hallucination, pleural effusion, and pneumonia that were commonly reported in the subgroup aged 18 and older were not observed in the subgroup of those aged younger than 18. On the other hand, cognitive disorder, febrile neutropenia, mental disorder, and gait disturbance were only reported in the subgroup aged younger than 18 (Table 4). Furthermore, some AEs such as death, dyspnoea, and pleural effusion were more likely to occur in adult patients. High risk AEs in pediatric patients included hydrocephalus, psychotic symptom, and liver function test increased (Table 5, Detail in Supplementary Table S2).

**TABLE 4:** Top 15 lorlatinib-related adverse events (AEs) based on age subgroup.

Age (<18)		Age (≥18)	
AE	Number	AE	Number
Off label use	28	Death	445
Neoplasm progression	16	Neoplasm progression	386
Cognitive disorder	10	Off label use	115
Death	9	Dyspnoea	104
Febrile neutropenia	9	Blood cholesterol increased	91
Hyperlipidaemia	9	Oedema peripheral	82
Hypercholesterolaemia	8	Weight increased	78
Weight increased	8	Hallucination	72
Hypertriglyceridaemia	7	Hypercholesterolaemia	69
Oedema	7	Peripheral swelling	63
Blood cholesterol increased	6	Pleural effusion	60
Mental disorder	5	Pneumonia	60
Wrong technique in product usage process	5	Oedema	55
Blood triglycerides increased	4	Blood triglycerides increased	53
Gait disturbance	4	Memory impairment	53

**TABLE 5:** Age-based subgroup analysis of lorlatinib-related adverse events (AEs).

AE	Age (<18)	Age (≥18)	Odds ratio
Death	9	445	4.03
Dyspnoea	1	104	7.35
Haemoglobin decreased	1	41	2.80
Oedema peripheral	2	82	2.84
Pleural effusion	1	60	4.14
Hydrocephalus	2	4	0.13
Hyperglycaemia	3	9	0.20
Leukoencephalopathy	1	2	0.13
Liver function test increased	2	4	0.13
Psychotic symptom	1	2	0.13

The top 15 AEs were generally similar between females and males. To be specific, blood triglycerides increased and memory impairment were found to be more frequently reported in females, while pneumonia and pyrexia were only reported in males (Table 6). In addition, generalized oedema, delusion, and language disorder were found to be more common in females. On the other hand, a higher incidence of blood urea increased, body mass index increased, and pleural thickening was observed in males compared to females (Table 7, Detail in Supplementary Table S3).

**TABLE 6:** Top 15 lorlatinib-related adverse events (AEs) based on gender subgroup.

Female		Male	
AE	Number	AE	Number
Death	271	Death	263
Neoplasm progression	227	Neoplasm progression	206
Off label use	101	Dyspnoea	55
Blood cholesterol increased	70	Off label use	54
Weight increased	58	Blood cholesterol increased	43
Oedema peripheral	55	Hallucination	42
Dyspnoea	53	Weight increased	40
Hallucination	53	Oedema peripheral	39
Hypercholesterolaemia	51	Pneumonia	37
Cognitive disorder	50	Hypercholesterolaemia	30
Oedema	48	Peripheral swelling	30
Peripheral swelling	41	Pleural effusion	29
Blood triglycerides increased	40	Oedema	25
Memory impairment	39	Cognitive disorder	23
Pleural effusion	38	Pyrexia	23

**TABLE 7:** Gender-based subgroup analysis of lorlatinib-related adverse events (AEs).

AE	Female	Male	Odds ratio
Cerebral disorder	5	1	4.11
Delusion	6	1	4.94
Generalised oedema	10	1	8.25
Ischaemic stroke	4	1	3.29
Language disorder	5	1	4.11
Blood urea increased	1	6	0.14
Body mass index increased	2	8	0.20
Lung opacity	1	4	0.20
Pleural thickening	1	5	0.16
Thrombophlebitis migrans	1	4	0.20

### Time to onset of lorlatinib-related AEs

**FIGURE 1:**
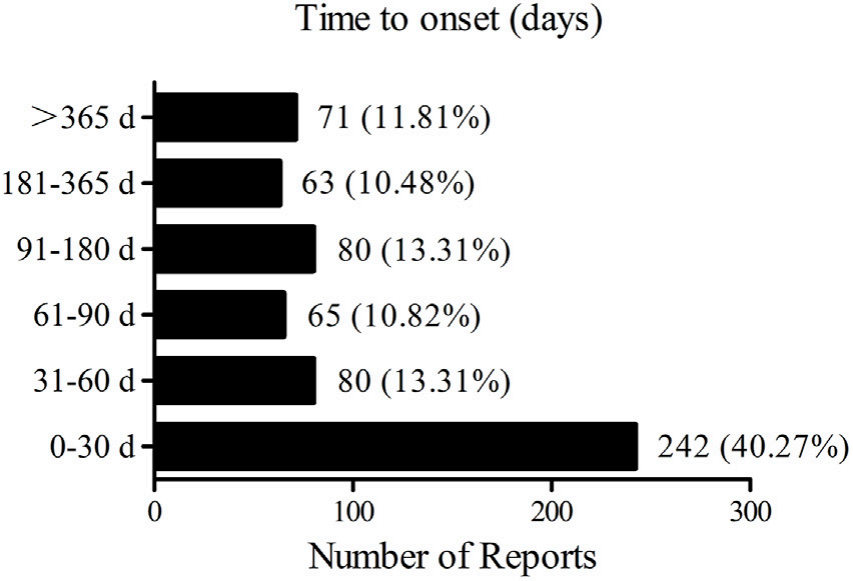
Time to onset of lorlatinib-related adverse events.

A total of 601 lorlatinib-related AEs with available onset times were included for analysis. The median onset time was 51 days (interquartile range [IQR] 13–152 days). As illustrated in Figure 1, more than half of the AE cases occurred within the first 1 (*n* = 242, 40.27%) and 2 (*n* = 80, 13.31%) months after lorlatinib initiation. Of note, a small proportion of AEs (*n* = 71, 11.81%) still occurred after 1 year of lorlatinib treatment possibly due to the long report period.

## Discussions

As a third-generation ALK-TKI, lorlatinib has a distinct safety profile that is different from those of other ALK-TKIs, including hyperlipidemia, peripheral neuropathy, and central nervous system AEs. In the present study, we conducted a comprehensive pharmacovigilance analysis of the post-marketing safety profile of lorlatinib based on the FAERS database. We identified new potential AEs related to lorlatinib, compared the age and gender differences, and evaluated the onset time of lorlatinib-related AEs. Our results may provide valuable information for the update of drug label and the rational use of lorlatinib in clinical practice.

In the present disproportionality analysis, the most common AEs including hypercholesterolemia, hypertriglyceridemia, oedema, cognitive effects, mood effects, and peripheral neuropathy were in line with the drug instructions and previous studies (Syed, 2019). Further analysis showed that death and neoplasm progression which were not recorded in the drug instruction were reported with the most cases. Similarly, progression of neoplasms in neoplasms was identified as the most frequently reported adverse event for lorlatinib in a recent post-marketing safety study (Omar et al., 2021). Moreover, no lorlatinib-related deaths were reported in previous studies (Shaw et al., 2017; Solomon et al., 2018). Therefore, the frequently reported death and neoplasm progression might be attributed to the characteristics of metastatic NSCLC itself or the patient disease progression rather than lorlatinib.

Lung cancer is associated with a high incidence of brain metastases. Lorlatib was designed to be CNS penetrant and indeed had shown marked intracranial activity in preclinical and clinical studies (Zou et al., 2015; Shaw et al., 2017). However, due to the high brain penetrance and resulting accumulation in the CNS, AEs in nervous system disorders (including memory impairment, cognitive disorder, and amnesia) and psychiatric disorders (including hallucination, confusional state, and affect lability) were frequently reported which were also confirmed in our study. Indeed, CNS AEs occurred in a significantly higher proportion of patients who received lorlatinb (35%) compared to patients who received crizotinib (11%) (Solomon et al., 2022). CNS AEs were mainly mild in severity and improved on dose modifications. Of note, cognitive effects were the most common AEs that led to permanent discontinuation (Yang and Gong, 2019). Therefore, patients should be advised to report any changes in cognitive function, mood, speech, and mental status during treatment with lorlatinib.

Interstitial lung disease (ILD) was a serious adverse event associated with ALK-TKIs with a global incident rate of 2.1% (Suh et al., 2019; Zhao et al., 2023). A recent pharmacovigilance study reported that lorlatinib was statistically associated with ILD and resulted in a high death rate (Zhao et al., 2023). Nevertheless, in the present study, we identified 9 cases of ILD without a positive signal (ROR 1.10, 0.57-2.12). The possible difference might be due to the different data extraction methods. In Min Zhao’s study, ILD was a standardized MedDRA query containing 45 terms at the PT level. However, we focused on the specific term of ILD which might provide a more accurate result. Moreover, our results identified pneumonia (ROR 1.28, 1.01-1.63) and pneumonitis (ROR 5.83, 4.02-8.46) as positive signals with 70 cases and 28 cases, respectively. Interestingly, a recent report presented a case of successful switch to lorlatinib treatment without ILD relapse after the onset of drug-induced ILD caused by alectinib (Myall et al., 2021; Kashizaki et al., 2022). Considering that ILD was indicated in the drug label, the association between lorlatinib and ILD and the safety and administration strategy of lorlatinib in clinical patients require further investigation.

Our analysis identified pulmonary arterial hypertension (PAH) as a positive signal associated with lorlatinib. Consistently, PAH was found to be significantly related to lorlatinib in a recent pharmacovigilance analysis using FAERS (Syed, 2019). Of note, Chabrol et al. (2018) reported that two patients with metastatic NSCLC developed severe PAH within 2 months after lorlatinib treatment, and dramatically improved after lorlatinib withdrawal. Although PAH is not included in the drug insert of lorlatinib so far, our results suggest that more attention should be paid to the potential risk of PAH during lorlatinib treatment and cardiovascular evaluation could be conducted before lorlatinib introduction.

Among the SOC of cardiac disorders, we found 7 significant PTs: 1 PT included in the label and 6 new unexpected PTs not included in the label. Interestingly, 4 new unexpected PTs (cardiac failure, pericardial effusion, cardiac tamponade, and cardiomyopathy) also had positive signals in a previous pharmacovigilance study that focused on the ALK-TKI-associated cardiotoxicity (Liu et al., 2022). It is worth noting that pericardial effusion and cardiac tamponade were life-threatening complications of NSCLC, and severe pericardial effusion was most likely caused by advanced cancer itself (Wang et al., 2000; Mufti et al., 2018). Moreover, the symptoms of pericardial effusion could be effectively improved by other ALK-TKIs (Gadgeel et al., 2014; Matsuo et al., 2016). Currently, the associations between lorlatinib and pericardial effusion and cardiac tamponade are not clear. In addition, we identified 3 cases of AV block, 1 case of AV block first degree, and 1 case of AV block second degree in the present study. However, none of them met the criteria for positive signals. Consistently, no positive signal of AV block was detected in previous pharmacovigilance analysis of FAERS data (Syed, 2019; Liu et al., 2022). In a phase I/II study, no AV block was reported in healthy volunteers who were administrated lorlatinib. However, of the 295 patients treated with lorlatinib, 2 cases of asymptomatic first-degree AV block and 1 case of complete AV block were observed. Moreover, the patient who reported complete AV block had pre-existing second-degree AV block (Bauer et al., 2019). Of note, the FDA drug instruction indicated lorlatinib-induced AV block, but not lorlatinib-induced cardiomyopathy. Our study suggested that further studies were required to confirm the cardiac AEs caused by lorlatinib.

For radiation necrosis, our analysis found a significant signal (ROR 78.00, 38.60-157.63) with lorlatinib though only 8 cases of radiation necrosis were identified. Viola et al. reported two cases of radiation necrosis developed shortly after lorlatinib initiation following progression on the sequential treatment of crizotinib, alectinib, and brigatinib (Zhu et al., 2020). Considering that radiation treatment and NSCLC were identified as risk factors for developing radiation necrosis (Miller et al., 2016; Vellayappan et al., 2018), there remains a possibility that lorlatinib might accelerate the development of radiation necrosis that pre-existed before lorlatinib treatment. Interestingly, Ou et al. (2016) reported a case that radiation necrosis occurred 7 years after the last radiation treatment and 1 year after alectinib introduction. The results of our study and several case reports indicated that radiation necrosis might be a potentially rare but clinically meaningful AE related to lorlatinib. Large-scale studies are needed to recognize whether radiation necrosis is the result of inherent tumor biology, radiation treatment, lorlatinib treatment, or a combination of these factors.

It is well known that age and gender have relevant impacts on the bioavailability, metabolism, and elimination of drugs, leading to different efficacy and AEs (Farkouh et al., 2020; van Kampen et al., 2023). Currently, the report of age-specific and gender-specific AEs related to lorlatinib is unclear. In the present study, we observed a markedly higher number of positive signals in the subgroup aged 18 and older compared to the subgroup aged younger than 18. Moreover, AEs such as pleural effusion and pneumonia were only identified in the subgroup aged 18 and older which might be due to the increasing incidence of pneumonia with age (Henig and Kaye, 2017). Interestingly, pneumonia was more commonly reported in males which might be associated with their longer airways compared to females (Talaminos Barroso et al., 2018). Our results would provide important information for detailed clinical management based on specific subgroup characteristics. Further studies are required to confirm the age and gender differences in the safety profile of lorlatinib for better drug regimens and optimal drug therapy.

Our results indicated that more than half of lorlatinib-related AEs occurred within the first 2 months after the lorlatinib medication with a median time to onset of 51 days. This was compatible with the results from previous studies that lorlatinib-related hyperlipidemia, CNS effects, weight increase, edema, and peripheral neuropathy occurred with a median time to onset of 15 days, 42 days, 64 days, 42 days, and 77 days, respectively (Bauer et al., 2019). In addition, 11.81% of lorlatinib-related AEs still occurred after 365 days. Therefore, the long-term safety of lorlatinib deserves more attention in future clinical studies.

There are several limitations in our current study. First, as the FAERS database is a spontaneous reporting system, potential reporting bias, under-reporting, and over-reporting probably exist. The AE information might be incomplete, inaccurate, and heterogeneous due to the different reporters (professionals and non-professionals). Further, the underlying confounding factors such as drug-drug interactions and patient medication history were unclear. Therefore, a causal relationship between lorlatinib and AE reports could not be established based on our disproportionality analysis. Second, though our present study provided the statistical association between lorlatinib and AEs, the incidence of AEs related to lorlatinib could not be calculated as the total number of cases that occurred in the population was unknown according to the FAERS database. However, our study provided a comprehensive analysis of the safety profile of lorlatinib in real-world settings. In addition, new unexpected AEs such as radiation necrosis were also identified in our study. Based on our results, a general framework of “prepare, monitor, manage, reassess” could be followed to provide practical, actionable, and personalized patient care. Clinicians should keep in mind the likelihood and nature of lorlatinib-related AEs and encourage patients to report any symptoms during lorlatinib treatment. Patients should undergo a series of baseline tests such as biochemical analysis, cardiovascular risk evaluation, neurological evaluation, and contrast-enhanced magnetic resonance imaging of the brain before lorlatinib treatment. Our study added valuable information for the evaluation of the safety profile of lorlatinib in clinical practice.

## Conclusion

In conclusion, the present study performed a comprehensive pharmacovigilance analysis of lorlatinib based on the FAERS database. AEs such as ILD and AV block indicated in the drug label require further evaluation. More attention should be paid to the new potential unexpected AEs including PAH and radiation necrosis. Additionally, the potential age and gender differences should be considered for optimal drug therapy in clinical practice. Our study provides valuable information for the rational and safe administration of lorlatinib in the clinic. Further prospective studies are required to confirm the associations between lorlatinib and the identified AEs.

## Data Availability

The original contributions presented in the study are included in the article/Supplementary Material, further inquiries can be directed to the corresponding author.

## Abbreviations

AE, adverse event; ALK, anaplastic lymphoma kinase; CI, confidence interval; CNS, central nervous system; EMA, European Medicines Agency; FAERS, Food and Drug Administration Adverse Event Reporting System; FDA, Food and Drug Administration; ILD, interstitial lung disease; IQR, interquartile range; MedDRA, Medical Dictionary for Regulatory Activities; NSCLC, non-small cell lung cancer; PAH, pulmonary arterial hypertension; PS, primary suspect; PT, preferred term; ROR, reporting odds ratio; SOC, system organ classes; TKI, tyrosine kinase inhibitor.
